# Challenges and solutions for the promotion of medical sciences faculty members in Iran: a systematic review

**DOI:** 10.1186/s12909-022-03451-2

**Published:** 2022-05-26

**Authors:** Mahla Salajegheh, Somayeh Noori Hekmat, Maryam Macky

**Affiliations:** 1 Health Services Management Research Center, Institute for Futures Studies in Health, Kerman University of Medical Sciences, Kerman, Iran; 2 Department of Medical Education, Education Development Center, Kerman University of Medical Sciences, Kerman, Iran; 3 Modeling in Health Research Center, Institute for Futures Studies in Health, Kerman University of Medical Sciences, Haft-Bagh Highway, Kerman, Iran; 4 Environmental Health Engineering, Department of Environmental Health, Kerman University of Medical Sciences, Kerman, Iran

**Keywords:** Iran, Faculty, Medical, Medicine

## Abstract

**Introduction:**

The faculty promotion system is expected to benefit the faculty, institute, and profession and lead to the sustainable and comprehensive development. This present systematic review aims to investigate the challenges and solutions for the promotion of medical sciences faculty members in Iran.

**Method:**

This study was a systematic review conducted by searching in PubMed, Scopus, Eric, Web of Science, Cochrane, SID, Magiran, and https://irandoc.ac.ir/line with Persian and English terms in the period from 2015 to 2020. Study selection and data extraction were performed independently by reviewers.

**Results:**

Thirteen articles were included. Challenges and solutions for the promotion of medical sciences faculty members were reviewed and grouped into five main categories: 1. The general regulations for the promotion of faculty members, 2. Cultural, disciplinary, and social activities, 3. Educational activities, 4. Research-technology activities, and 5. Scientific-executive activities.

**Conclusion:**

Despite several modifications to regulations for the promotion of medical sciences faculty members in Iran, this process still encounters challenges because of its complex nature. This article provides tips to policymakers on regulations of promotion for educational activities.

## Background

As an educational center, a medical sciences university needs committed human resources with special skills and knowledge to achieve its goals. One of the main components of any medical sciences university are faculty who are responsible for training students [1]. Therefore, recruiting and employing capable faculty members, motivating them, and promoting their professional lives are vital to enhance the efficiency of medical education institutions [2]. The regulations for faculty members promotion in medical universities play a substantial role in leading the faculty’s activities and directing policy-making for higher education [3]. The promotion system is one of the most important aspects which affecting the performance of each faculty at the medical universities [4]. These regulations should aim at guiding the faculty for sustainable and comprehensive development [5]. Successful promotion benefits the faculty, institute, and profession [6, 7]. In fact, there is a critical connection between academic development and academic promotion [8].

In different countries, some studies have been conducted on faculty member promotion criteria, structure, and processes. Gardner et al. (2013) discovered issues of time, lack of clarity, and gender disparity concerning faculty members who promoted to full professor rank [9]. Eckhaus et al. (2019) revealed that the faculty found an association that causes harm to their promotion processes as a result of student evaluations [10]. Despite the important role of the academic promotion, evidence show various obstacles to promotion for faculty.

In this way, some of the results showed that the process of faculty promotion in Iran is a stressful process [11]. However, these studies have mainly considered the evaluation and the promotion of faculty members without especially identifying challenges nor providing solutions. So, due to the ambiguities and complexities surrounding faculty member promotion, there is a need to conduct a comprehensive study to look into the various aspects of this issue in more detail. To our knowledge, no systematic review has been published in the regard of challenges and solutions for the promotion of medical sciences faculty members in Iran.

Since policymakers of higher education seek evidence to improve the individual and collective capacities of the higher education institutions, informing their future planning, and considering best possible resources to reinforce or modify the subsequent educational process, these results will capable of capturing the complexities of promotion of medical sciences faculty members. In this regard, the purpose of this systematic research was to investigate the challenges and solutions for the promotion of medical sciences faculty members in Iran.

## Method

This was a systematic review exploring the challenges and solutions for the promotion of medical sciences faculty members in Iran. The researchers assessed all the findings related to the criteria required for evaluating faculty member tasks including cultural, social, educational, research-technology, and scientific-executive activities. This study was performed based on the Preferred Reporting Items for Systematic Reviews and Meta-Analyses (PRISMA) statement to ensure the high quality and answer some questions about the challenges in faculty member promotion regulations and provide appropriate solutions [12]. This study was approved by the Research Ethics Committee of Kerman University of Medical Sciences (No. IR.KMU.REC.1400.642).

### I. Search strategy

A preliminary list of terms was compiled after an initial review of relevant studies and consultation with experts. A rapid search was carried out using the preliminary list of terms. Then, by reviewing the titles and abstracts of the articles retrieved in the rapid search, the list of terms was finalized, and the SPIDER table was produced (Table 1).

**Table 1:** SPIDER search strategy

SPIDER	Keyword	Synonyms			
Sample	Faculty member	Faculty	Professor	Academic Member	
Phenomenon of Interest	Tenure	Appointment	Maintenance	Recruitment	Promotion
Evaluation					

The search was conducted throughout 2015 to 2020. We performed the search in this period of time because the current regulations which is now using for the promotion of the medical sciences faculty members in Iran was updated since 2015. The search was done in PubMed, Scopus, Eric, Web of Science, Cochrane, SID, Magiran, and https://irandoc.ac.ir/line. Also, the websites of journals interested in medical sciences education, including Medical Education Journal, Strides in Development of Medical Education Journal, Hakim Research Journal, Payesh Quarterly, Journal of Health Management and Teb va Tazkiye Quarterly, were also searched. Google Scholar was also involved for more comprehensiveness. A manual search was performed using Backward and Forward Reference Searching to further complete the search strategy. The references of the included articles were reviewed through backward tracing to access the most relevant articles published in previous years, while forward tracing was useful to retrieve articles included in the study. Experts were consulted and the publications on the relevant websites were searched to find out gray literature.

### II. Inclusion and exclusion criteria

Studies whose purposes were in line with the research question and were published in Persian or English were included. Studies which described the faculty promotion regulations for non-medical sciences universities were excluded.

### III. Quality assessment of the studies

We used the BEME checklist [13], including of 11 indicators, to assess the quality of studies. Each indicator was rated as “met,” “unmet,” or “unclear.” In order to be deemed of high quality, articles should meet at least seven indicators. The quality of the full text of potentially relevant articles was assessed by one author and checked by the second author (MS and MM). Disagreements were fixed through discussion. No study was removed based on the results of quality assessment.

### IV. Data analysis

After removing the duplicates, each study potentially meeting the inclusion criteria was independently screened by the two authors (MS and MM). The most related titles were selected, then, the extracted articles were screened for their abstracts. In case of relevance, the full texts were investigated. The full texts of articles were reviewed and coded simultaneously by two researchers, then, they were entered into the MAXQDA 10.2 software. Coding has been done using the inductive approach to extract the findings. To ensure that all the codes were reviewed in the initial stage, the studies were re-reviewed and compared against the final list of codes.

## Results

**Fig. 1:**
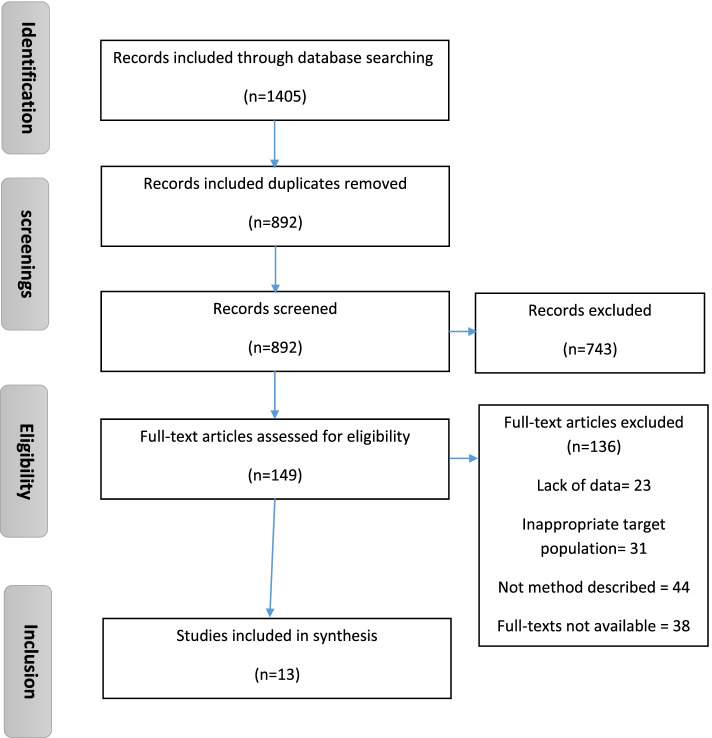
Flowchart of the selection steps

Initially, 1405 articles were identified. In the screening stage, 513 articles were excluded in the screening because of duplication, and 743 articles were deleted by matching the titles and abstracts with the inclusion criteria. In the eligibility stage, 149 articles were assessed by reviewing the full texts. Because of reasons including lack of data, inappropriate target population, not describing method, and full texts not available, 136 articles deleted after reading the full texts. Eventually, 13 articles were included in the study. Of these, 8 were published in Persian, and 5 were in English. The PRISMA diagram for included studies is shown in Fig. 1. The characteristics of the included articles are presented in Table 2.

**Table 2:** Characteristics of the included articles related to the faculty member promotion regulations

Number	First author	Year Published	Journal	Material reviewed
1	Hossein Karimi-Moonaghi	2015	Journal of Educational Development in Medical Sciences	General content of the regulations
2	Abdolreza Gilavand	2016	International Journal of Medical Research & Health Sciences	General content of the regulations
3	Shannon B Smith	2016	Journal of Professional Nursing	Research-technology activities Educational activities
4	Samaneh Ebrahimpour	2017	Social Welfare Quarterly	Scientific-executive activities Educational activities
5	Batool Jamali Zavareh	2018	Iranian Higher Education	Research-technology activities Educational activities Cultural activities
6	David Moher	2018	PLoS biology	Educational activities
7	Elaheh Abolhoseini	2018	Archives of Rehabilitation	Research-technology activities
8	Fariba Nasiri Ziba	2018	Paramedical Sciences and Military Health	Educational activities
9	Mehdi Mohammadi	2018	Research in Medical Education	General content of the regulations
10	Susan M McHale	2019	Journal of Clinical and Translational Science	Research-technology activities Educational activities
11	Seyedeh Susan Raoufi Kelachayeh	2020	Journal of Health Promotion Management	
12	Meredith T. Niles	2020	PLoS One	Educational activities General content of the regulations
13	Patricia C Clark	2020	Journal of professional nursing	Research-technology activities Educational activities

Content analysis of the articles related to the regulations for the promotion of faculty members was carried out based on five perspectives: 1. the general content of the regulations for the promotion of faculty members; 2. cultural, disciplinary, and social activities, 3. educational activities, 4. research-technology activities, and 5. scientific-executive activities. The relevant codes were compiled according to Table 3.

**Table 3:** Codes relevant to the faculty member promotion regulations

Main category	Subcategory	Codes
The general content of the regulations for the promotion of faculty members	Challenges to the general content of the regulations for the promotion of faculty members	• Low emphasis on innovation and creativity and dominance of the quantitative attitude • Failure to pay attention to the differences between universities and disciplines • Weakness in modeling global experiences • The inefficiency of control structures of faculty’s scientific recession • Administrative function instead of focusing on the comprehensive promotion of education, research, and scientific and cultural services • Difficulty in measuring abstract concepts • Failure to respond to the conditions of specific groups (women, general education groups)
	Solutions for the general content of the regulations for the promotion of faculty members	• Changing the University Board of Assessors periodically • Establishment of a consulting and facilitation unit for the preparation of the promotion’s documents • Implementing symposiums to exchanging views between the supervisory boards of different universities • Close monitoring of the assessment committees over the performance of the selected faculty committees • Setting rules governing the executive process of reviewing promotion cases • Supervising the composition of distinguished board members (diversity of fields of study, presence of women in these boards, different academic degrees) • Developing appropriate laws to reduce conflicts of interest
The cultural, disciplinary, and social activities	Challenges to cultural, educational and social activities	• Lack of transparency in the indicators of cultural activity and ambiguity in scoring them • Narrowing cultural activities to participation in specific educational courses • Lack of reflection of priorities for changing organizational and social culture • Neglect of some cultural activities related to the Comprehensive Plan and Islamization of Universities Document • Neglect of the development and promotion of the humanities
	Solutions for cultural, disciplinary, and social activities	• Creating the necessary facilities for cultural activities • Setting criteria for awareness of faculty members’ abilities, capabilities and interests • Providing facilities for scientific and professional servicing to the public • Playing a role in programs related to promoting security or environmental protection and convergence of education and research with moral and spiritual education at universities
Educational activities	Challenges to educational activities	• Confrontation of educational and research activities instead of reinforcing each other • Limiting educational activities to the number of required teaching units • Homogeneity and use of identical tools and forms of assessment • The inefficiency of teaching quality evaluation systems
	Solutions for educational activities	• Attention to the breadth and variety of educational activities • Emphasis on the use of new educational technologies • Emphasis on education based on up-to-date and valid science • Utilizing a combination of quantitative and qualitative evaluation methods and using multiple resources • Matching a particular share of promotion indicators with the mission, requirements, special conditions, scientific resources and facilities of each university of medical sciences • Assessing the role of the individual in promoting the relevant department • Allocation of points for activities related to social accountability and community education
Research-technology activities	Challenges to research-technology activities	• Significant emphasis on research activities compared to other activities • High emphasis on science production in the form of ISI papers • Encouraging faculty members to produce papers regardless of the needs of the society • Paying attention to quantity instead of quality in papers • The complex situation of commercialization and knowledge production • Inequalities in the use of grants and research funds
	Solutions for research-technology activities	• Orientation towards meeting the research needs of the society • Looking at research activities from the perspective of a teacher and not just from the perspective of research as an entity separate from education • Encouraging the absorption of research funding from outside the university • Emphasis on following a specific research line • Evaluating the quality of the articles by an impartial expert team • Emphasis on convergence and interdisciplinary activity • Assigning scores to new ways of disseminating knowledge • Playing a role in advancing and creating change in the relevant scientific field • Introducing scientific fields to the society in the relevant scientific ground
Scientific-executive activities	Challenges to scientific-executive activities	• Ease in providing executive privileges and reducing their effectiveness in encouraging faculty members to accept executive responsibility • Ignoring the social status of faculty members • Ignoring the tension and stress caused by executive responsibilities • Ignoring the quality of one’s performance in executive responsibility • Ignoring the lower chances of women in holding executive positions compared with men
	Solutions for scientific-executive activities	• Emphasis on the quality of executive responsibility • Playing a role in facilitating and promoting the functions and achieving the goals of the university

### General content of the regulations for the promotion of faculty members

According to the most articles reviewed, regulations for promoting faculty members restrict their creativity and interests. In other words, the regulations are more oriented towards an administrative function rather than focusing on the comprehensive promotion of faculty members [14–16].

Another challenge to the regulations is that they consider similar conditions for all universities, disciplines and individuals [17, 18]. In fact, there are various needs in the disciplines and also potential capabilities of each area of the country is different. But the regulations assess all these disciplines, universities, and individuals based on the same structure [19]. Moreover, the mission, requirements, special circumstances, resources, and scientific facilities of each university have not been considered.

In addition, the difficulty in measuring abstract concepts and the requirement for faculty members to gain score in all categories are other shortcomings in this regard [11, 20].

Some issues such as lack of transparency, absence of specialized staff in promotion committees, long-term process, unnecessary administrative requirements, and the conflict of interest may be seem as the other challenges [21]. Probably, changing the University Board of Evaluators periodically, close supervision and monitoring them, and establishing an advisory unit to guide and help the applicant faculty for the promotion can address these challenges [22].

### Cultural, disciplinary, and social activities

Cultural, disciplinary, and social activities of faculty members are crucial as these members act as role models for their students and society. Few studies have examined these activities, and their results indicate challenges such as lack of transparency in guidelines and rules in evaluating cultural, disciplinary, and social activities and lack of knowledge in faculty about these activities [23]. Proposed solutions for addressing these challenges are including providing the necessary facilities for cultural activities and paying attention to the abilities and interests of each faculty members. Also, acknowledgment the convergence of education and research with moral and spiritual education at universities and providing opportunities for this aspect may be consider as a solution [24].

### Educational activities

Due to the vital role of faculty members in universities, the educational activities in the promotion regulations are intrinsic. But most of the results revealed significant challenges in the educational part of promotion regulations. In this regard, we can point out to the prominent number of mandatory teaching credits. The quantity of teaching in the promotion process reflects only the faculty member’s presence in the classroom, and the quality of education is seldom considered. As another challenge, the publication of scientific papers has become a daily concern for faculty members. This leads to decrease the amount of time spent on educational activities and executive responsibilities [18]. Some solutions that can be proposed are including emphasis on employing new methods of teaching and assessment, using more appropriate instructional materials, participation in educational faculty development programs, cooperation in the curriculum development or revision, production of educational materials, and activity in the field of educational management and leadership [11].

Other challenges in educational activities include inefficiency of methods for teaching evaluation [19, 22, 23]. In order to moderate these challenges, more attention ought to be paid to the quality of teaching evaluation by involving different sources and methods of gathering data [11].

### Research-technology activities

In spite of the importance of research in improving the performance of universities, some challenges to research-technology activities which prevent the useful application of the potential results of faculty members’ research efforts. The regulations in this category lead faculty to simply produce papers without considering the actual needs of society [11]. Also, focusing on the number of papers instead of quality of them has adversely reduced other research activities such as writing and translating books [15]. Some of the findings revealed that increasing the sustainability and destination of research activities and emphasizing on originality and innovation, are some suggestions to reform regulations of research activities [18].

### Scientific-executive activities

Challenges to scientific-executive activities are including lack of interest in accepting executive responsibilities in the university, ignoring the social impact of faculty members activities, and limited chance available to female faculty to occupy managerial positions [25]. Applying strategies such as raising the quality of administrative work, facilitating the functions of the university to achieve its goals can contribute to solve the above challenges [26].

## Discussion

This is the first systematic review highlighted the challenges and solutions for the promotion of medical sciences faculty members in Iran. One of the critical aspects to maintain the quality and efficiency of higher education is the system of faculty member promotion [24, 25]. Based on the results of the reviewed studies, the current criteria of faculty member evaluation lack the ability to depict the quality of faculty members’ efforts and render a comprehensive analysis of their performance [26, 27]. Besides, faculty members have opposed the assessment techniques utilized by the evaluation boards as they generally depend on personal favoritism, slowness of the process, and some cases of injustice [28–30]. The promotion of faculty members should be based on an accurate and impartial evaluation to increase their motivation and job satisfaction [31–33]. In this regard, some studies have pointed out the need for developing different regulations for the promotion of faculty [34, 35].

One of the solutions for the challenges related to the general contents of regulations is to design and implement faculty development programs about the promotion regulations. These programs impact faculty members at individual and organizational abilities [36, 37], and lead to increase their awareness about the promotion process.

Because of the system governing universities of medical sciences in Iran, cultural and educational activities are mainly considered, and all stakeholders agree on the need to pay attention to these activities. However, challenges related to the abstractness of these concepts and the difficulty of measuring them in the form of academic activities have resulted in negative attitudes towards cultural activities among faculty. The results of previous studies which show a negative attitude towards cultural activities [35] and the inevitable need to develop both appropriate qualitative and quantitative indicators to measure these activities are consistent with the results of our research [33].

The existence of many challenges to educational activities is an alert for policymakers of the higher education promotion system. As reported in some studies, one of the main concerns of faculty members is the lack of attention to the quality of education [38, 26]. Therefore, the educational activities need to evaluate from a qualitative perspective and direct towards innovation, teamwork, and inter professional activities which ultimately aim to improve the organizational development [39, 40].

Due to the value of research in addressing public concerns, it is necessary to direct the relevant activities of faculty members towards responsiveness the needs of society, creating change, advancing the scientific field, and engagement in the national policymaking process [38]. Qualitative review of a limited number of faculty members’ papers would draw more attention to the quality of research instead of concentrating on increasing the number of papers [39].

Although the regulations have generally specified their approach as one that serves the society, in most cases, faculty members deviate from this goal and pursue more executive positions that are far detached from the real needs of society [41]. In fact, the existing system of faculty promotion has an incorrect alignment with the needs of society and is disconnect from the reproducibility of the medical universities [27]. Determining specific criteria in this respect would help faculty members to further focus on improving the quality of the university’s performance in achieving its scientific, disciplinary, and cultural mission. This would assist universities to play a core role in policymaking and service to their society [41]. The regulations should also guide the evaluation of executive scientific activities so that faculty members can place their abilities and knowledge in the service of society in various ways.

Some of the challenges that reported in this review are compatible with the past researches about the academic promotion rules. Dhulkhed et al. [42] discussed that the academic promotion regulation in India has the potential to decrease the quality of teaching and learning process and lead to most effort of faculty be on the research publication to fulfill the promotion criteria. They argued an urgent need to revise the current promotion criteria based on the comprehensive studies in this field. Also, Janjua et al. [43] in exploring the perceptions of faculty regarding the existing promotion criterion in Pakistan reported shortcomings such as unrealistic, inconsistent and biased academic promotion rules and lack of a justified and faired faculty evaluation process.

Due to the absence of a systematic review on challenges and solutions facing the process of promotion of medical sciences faculty members in Iran, one of the strengths in this study is the comprehensive review of all aspects of the promotion regulations. These findings provide guides for educational policymakers to improve the promotion process of medical sciences faculty members in Iran and also the leading countries in science. The information paucity in some articles was as a limitation in the present research.

## Conclusion

Reviewing the system of medical sciences faculty member promotion will result in more dynamic education system, promoting the scientific level at universities, and ultimately improving social life. The results of this study will aid as a foundation for creating best practices and redesigning the existing approaches to assessing faculty members.

## Data Availability

The datasets used and/or analyzed during the current study available from the corresponding author on reasonable request.
